# An analysis of the role of college students' core self-evaluation in the relationship between extracurricular physical exercise and academic stress

**DOI:** 10.3389/fpsyg.2024.1279989

**Published:** 2024-02-27

**Authors:** Guanren Zhu, Wenwen Cao, Yutong Yin, Shengchao Bai

**Affiliations:** ^1^School of Physical Education, Huaiyin Normal University, Huaian, China; ^2^Department of Physical Education, Nanjing University of Science and Technology, Nanjing, China

**Keywords:** extracurricular physical exercise, academic stress, core self-evaluation, mental health, college students

## Abstract

**Objective:**

To explore the role of college students' core self-evaluation in the association between extracurricular physical exercise and academic stress, and to provide a reference and basis for effectively alleviating current college students' academic stress.

**Methods:**

A stratified cluster random sampling method was used to conduct an online survey using the China College Student Mental Health Screening Scale, the Core Self-Esteem Scale, and a self-developed questionnaire for 1,249 college students from 8 junior colleges in Jiangsu Province, China, in January 2023, and analyses were conducted using bivariate correlation and mediation effect tests.

**Results:**

More than half of the students were worried about lagging behind other students in their studies and worrying about exams, as well as more than half of the students had <2 h of extracurricular physical exercise per week in terms of academic stress. Significant correlations were found between extracurricular physical exercise time and core self-evaluation (*r* = 0.225, *p* < 0.01), academic stress (*r* = −0.317, *p* < 0.01), and between core self-evaluation and academic stress (*r* =−0.371, *p* < 0.01). Extracurricular physical exercise had a negative predictive effect on academic stress (*effect* = −0.306, 95%CI: −0.361~-0.251) and partially through the mediating path of core self-evaluation, (*effect* = −0.067, 95%CI: −0.091~-0.046), with a mediating effect of 21.9%. Among them, the mediating effect of core self-evaluation was highest in “worrying about lagging behind other students in their studies” and “worrying about exams”, which accounted for 24.4% and 24.3% respectively.

**Conclusion:**

Currently, college students have different degrees of academic stress; extracurricular physical exercise can reduce academic stress through direct effects, and also through the indirect effect of improving the level of core self-evaluation, and active extracurricular physical exercise has become an important way to promote students' physical and mental health.

## 1 Introduction

With the gradual expansion of higher education in recent years, college students have become one of the most stressful groups in Chinese society (Liu, 2009), with academics being a common stressor in campus life (Li et al., 2005). Academic stress, as a common problem faced by college students, has become a topic that has attracted widespread attention. More and more studies have shown that excessive academic stress can cause high blood pressure, cardiovascular disease and other physiological problems, but also lead to anxiety and depression and other psychological problems, and even suicidal tendencies (Nakalema and Ssenyonga, 2014). Academic stress has become an important factor affecting the physical and mental health of students in colleges (Garces-Arilla et al., 2023). Many studies have shown that physical exercise, as a positive psychological coping method, is believed to improve the body's immune system (Brolinson and Elliott, 2007), improve students' physical health, also antagonize psychological stress and channel psychological pressure (Mikkelsen et al., 2017). This is of great significance in alleviating the current academic stress of students.

The core self-evaluation of college students as a personality trait plays an important role in an individual's psychological coping and emotional regulation in the face of stress, which may play a key role in the relationship between physical exercise and academic stress. Core self-evaluation is an individual's basic self-evaluation and value judgement of self and external environment. It has been noted that there are significant differences in core self-evaluation levels among groups of college students, with students with high core self-evaluation having higher levels of self-esteem, more internal control and emotional stability, and higher self-efficacy (Paloş et al., 2023). Some studies have pointed out that core self-evaluation has a direct negative effect on academic stress and physical exercise is associated with all four core self-evaluative trait indicators, including self-esteem (Sonstroem and Morgan, 1989), self-efficacy (Wang et al., 2022), emotional stability (Morgan and Goldston, 1987), and sources of psychological control (Johnson et al., 2008). Among other things, increased physical exercise significantly increased students' levels of self-esteem and self-confidence (Liu et al., 2021), positively predicted self-efficacy (Gong et al., 2023), as well as increased recognition of their own abilities, which suggests that increased physical exercise improves core self-evaluation. Therefore, students' core self-evaluation may play an important role in mediating the relationship between extracurricular physical exercise and academic stress.

However, the theoretical framework of the relationship between academic stress, physical exercise, and core self-evaluation has not been clearly articulated in existing research, and in particular, the mediating mechanism of core self-evaluation between academic stress and physical exercise is rarely mentioned. Therefore, this study intends to analyze the current situation of extracurricular physical exercise and academic stress among college students through a cross-sectional survey, and on the basis of clarifying the relationship between the two, to conduct an in-depth investigation into the mechanism of core self-evaluation through the mediation effect test, aiming to explore the relationship between the three variables and to emphasize the mediating role of core self-evaluation between academic stress and extracurricular physical exercise, so as to provide theoretical references and practical bases for the targeted alleviation of current students' academic stress.

### 1.1 Concepts of extracurricular physical exercise, academic stress, and core self-evaluation

#### 1.1.1 Extracurricular physical exercise

Extracurricular physical exercise refers to behaviors that enhance physical health and psychological wellbeing through participation in various extracurricular physical exercise (Chen et al., 2019). Extracurricular physical exercise is believed to reduce negative emotions such as anxiety and depression, and enhance an individual's sense of self and wellbeing.

#### 1.1.2 Academic stress

Academic stress refers to the emotional and cognitive tension that individuals experience during the learning process due to factors such as heavy academic tasks and competitive pressures (Rahal et al., 2023). Academic stress not only affects an individual's psychological health, but also may lead to decreased interest in learning and poor learning outcomes.

#### 1.1.3 Core self-evaluation

Core self-evaluation is an individual's overall evaluation of his or her own values, abilities, and attitudes (Liu et al., 2023). It includes self-esteem, self-efficacy, emotional stability, and sources of psychological control, and has an impact on an individual's emotional regulation and coping ability.

### 1.2 Relationship between physical exercise and core self-evaluation

Physical exercise can enhance core self-evaluation by boosting positive experiences in self-esteem, self-efficacy, emotional stability, and sources of psychological control (Ferguson et al., 2023). By actively engaging in physical exercise, college students can overcome physical challenges, can clearly feel their ability to improve and value, and feel that they have the level of perseverance and overcoming difficulties, thus enhancing their self-esteem (Huang et al., 2023). When college students participate in regular physical exercise, they gradually improve their exercise level, experience their own progress and growth, and increase their confidence in their own abilities. This positive exercise experience can be transformed into a sense of self-efficacy in other domains, which in turn promotes the enhancement of core self-evaluation. Meanwhile, physical exercise can release the tension accumulated in the body (Becerra et al., 2022), and promote the balance of the endocrine system, thus improving the emotional state of the individual. During the exercise process, the individual may experience positive emotions such as happiness, relaxation, and pleasure, which can enhance the emotional stability in the core self-evaluation (Machado et al., 2019). In addition, there is a strong link between physical exercise and psychological control sources, and college students can positively influence the perception and coping styles of individual psychological control sources by enhancing the sense of internal control, improving coping ability, reducing external dependence, and enhancing psychological stress resistance (Marsh, 1992). It can be seen that physical exercise can improve the comprehensive perception of oneself through positive experiences, enhance core self-evaluation, and can penetrate into other areas of life, and this positive influence can help to improve psychological health, enhance the ability to resist setbacks, and enable individuals to better cope with various pressures and challenges (Guilmette et al., 2019).

### 1.3 Relationship between core self-evaluation and academic stress

Core self-evaluation plays an important role in regulating and influencing individuals' coping with academic stress. Self-esteem and self-efficacy in core self-evaluation are important components of an individual's evaluation of his or her own abilities and values. Students with higher self-esteem and self-efficacy tend to be more confident in coping with academic challenges and pressures (Chemers et al., 2001), face difficulties more positively, and are less susceptible to external negativity and negative influences. Conversely, individuals with lower self-esteem and self-efficacy may be more likely to feel depressed and powerless due to academic stress (Kenechukwu, 2018). In terms of emotional stability in the core self-evaluation, students with higher emotional stability tend to maintain a positive and optimistic emotional attitude in the face of academic stress, and are less likely to fall into an emotional trough, which helps to stabilize their psychological state. Students with strong self-control can better manage their emotions and learning strategies, thus reducing academic stress (Li et al., 2022). Meanwhile, individuals with stronger adaptive ability can more flexibly cope with different academic challenges and changes, and are less likely to experience excessive emotional fluctuations due to stress. In addition, in terms of the source of psychological control, students' self-perception and goal orientation will affect their feelings and coping styles of academic stress, and a positive sense of internal control will make individuals pay more attention to their own growth and progress, so that they can cope with academic stress more effectively (Özer et al., 2016). It can be seen that there is a complex and close relationship between core self-evaluation and academic stress. Different dimensions of core self-evaluation affect individuals' feelings, coping styles, and psychological regulation processes of academic stress. Therefore, exploring ways to improve core self-evaluation can help students better cope with academic stress and promote psychological health.

To summarize, the impact of academic stress on college students' mental health should not be ignored, which may lead to anxiety, depression and other adverse emotions. Physical exercise, especially extracurricular physical exercise, is considered to be a way to relieve academic stress, which helps to release tensions and enhance emotional state, and also promotes physical health. The relationship between academic stress and extracurricular physical exercise may be influenced by a number of factors. Core self-evaluation, as an individual's overall evaluation of himself or herself, including elements such as self-esteem, self-efficacy, emotional stability, and sources of psychological control, has attracted extensive attention in psychological research in recent years. Academic stress and extracurricular physical exercise have been recognized as two important factors in studies of college students' mental health. However, the relationship between the two is not a simple correspondence, and the mediating role of core self-evaluation in them has gradually become a topic that requires research attention.

## 2 Materials and methods

### 2.1 Objects

Using stratified cluster random sampling method for the study, four undergraduate and four specialized colleges were selected in January 2023 in Jiangsu Province, China, and stratified by grade in each school, and the random number table method was used to select classes in each grade, with physical education teaching classes as the sampling unit, and colleges that do not have physical education classes in the third and fourth grades were based on the physical education teaching classes of the second semester of the second grade, and all the students of the classes that had been drawn were entered into the survey. A total of 1,249 questionnaires were collected, and 1,108 valid questionnaires were returned, with a validity rate of 88.71%. The valid questionnaires included 646 undergraduate students, 462 specialized students, 572 male students, 536 female students, 309 freshmen, 325 sophomores, 337 juniors and 137 seniors. The inclusion criteria were: current students with normal cognitive level and voluntary participation in this survey. The questionnaire of this study was approved by the ethical approval of the Academic Committee of the Department of Physical Education of Nanjing University of Science and Technology (TYB-2023001), and informed consent was obtained from all the investigated students.

### 2.2 Methods

#### 2.2.1 Questionnaire survey

The survey included four aspects: the first part was the students' basic information, including gender (male = 1, female = 2), grade (1st grade = 1, 2nd grade = 2, 3rd grade = 3, 4th grade = 4), type of school (undergraduate = 1, specialist = 2), household registration (urban household = 1, rural household = 2), only child (yes = 1, no = 2), and other basic information Situation. The second part is the explanatory variable, which is academic stress, adopting the academic stress indicators in the China College Student Mental Health Screening Scale, which are four sub-indicators, namely, “I feel that I have difficulty in learning”, “I worry that I am lagging behind other students in studies” and “I cannot keep up with my studies even though I have put in a lot of effort”, “I worry about the examinations”, etc. Each item is assigned a value of 1 to 4 points, ranging from “not at all like me”, “not too much like me”, “more like me”, “very much like me”, and the higher the cumulative value, the greater the academic stress. The scale has been validated and used in many large-scale studies in China, which showed good validity, while the cronbach alpha coefficient in the questionnaire was 0.861, indicating that the reliability could satisfy the research analysis. The third part is the explanatory variable, which is extracurricular physical exercise time. Referring to the way of dealing with similar questions in China's official large-scale survey questionnaire “China Education Tracking Survey Data”, the question is set as “How many times of extracurricular physical exercise per week on average in the current semester? And how long is the duration of each time? (the autumn semester of 2022-2023)”, The criteria and basis for calculating overall weekly exercise time is: number of exercises × time per exercise = total exercise time. The limit values of ≥28 h total weekly exercise time were excluded from the recovered results, and the rest were included in the valid questionnaires. The fourth part is the mediator variable, for the core self-evaluation, this evaluation scale is a psychometric tool developed by Judge et al. to assess an individual's core self-evaluation. After its introduction to China, scholars translated and revised the scale to make it more consistent with the Chinese cultural context, and the revised version contains 12 declarative items covering four dimensions: self-esteem, self-efficacy, emotional stability, and sources of psychological control. This study uses a mature version that has been revised several times by Chinese scholars, the scale consists of 12 questions with 5-point Likert scoring and reverse scoring for the even numbered questions, the cronbach alpha coefficient in the questionnaire was 0.888, the reliability can be used for the research analyses.

#### 2.2.2 Quality control

In the design stage, the questionnaire gives priority to the widely used “China College Student Mental Health Screening Scale” and “the Core Self-Evaluation Scale”, and in the self-developed part, the questions are set up in the way of the large-scale survey data in China to ensure that the overall questionnaire can obtain high reliability and validity. The content of the questionnaire was optimized on the basis of the pre-test. Data entry was done in parallel using double-blind rules to ensure the credibility of the data. Hypothesis testing was performed during data processing in strict compliance with statistical requirements and all results were retained for review.

### 2.3 Statistical methods

After data entry, descriptive statistics were used to analyze the status quo of college students' extracurricular physical exercise time and academic stress; bivariate correlation analyses were used to analyze the interrelationships among extracurricular physical exercise time, academic stress, and core self-evaluation; and PROCESS was used, with the sample size of 5,000 set in bootstrap, to explore the mediating role of college students' core self-evaluation in extracurricular physical exercise time and academic stress, where statistical significance was judged by 95% confidence interval without zero, and the amount of mediating effect was calculated by the indirect effect as a proportion of the total effect. The overall statistical test level was α = 0.05.

## 3 Results

### 3.1 Descriptive analysis of college students' extracurricular physical exercise time and academic stress status

The students' weekly extracurricular physical exercise time averaged 2.351 ± 2.176 h. Among them, 34 students, or 3.1%, hardly exercised; a total of 350 students, or 31.5%, exercised for <1 h; a total of 646 students, or 58.3%, exercised for <2 h; a total of 920 students, or 83%, exercised for <3.5 h (an average of 30 min per day); and a total of 188 students, or 16.9%, spent more than 3.5 h per week on extracurricular physical exercise.

The average score of students' overall academic stress is 9.371 ± 2.507. Among them, the total number of students who “feel difficult in learning” is “more like me” and “very much like me” is 276, accounting for 24.9%; the total number of students who “worry about lagging behind other students in studies” is “more like me” and “very much like me” is 563, accounting for 50.8%; the total number of students who “not being able to keep up with the pace of study despite great effort” is “more like me” and “very much like me” is 240, accounting for 21.7%; the total number of students who “worry about the examinations” is “more like me” and “very much like me” is 573, accounting for 51.7%.

The specific status of college students' academic stress and extracurricular physical exercise time is detailed in Table 1.

**Table 1:** Subgroups of college students' academic stress and extracurricular physical exercise time.

**Variant**	**Subgroups**	**Extracurricular physical exercise time**	**Core self-evaluation**	**Total indicators of academic stress**	**Feeling difficult in learning**	**Worrying about lagging behind other students in studies**	**Not being able to keep up with the pace of study despite great effort**	**Worrying about exams**
Gender	Male	2.500 ± 2.214	43.829 ± 8.806	8.980 ± 2.525	2.016 ± 0.701	2.387 ± 0.883	2.133 ± 0.744	2.444 ± 0.920
	Female	2.193 ± 2.128	43.323 ± 8.902	9.791 ± 2.421	2.157 ± 0.693	2.610 ± 0.881	2.256 ± 0.751	2.7695 ± 0.927
Only child	Yes	2.610 ± 2.497	43.649 ± 9.085	9.215 ± 2.403	2.063 ± 0.682	2.568 ± 0.823	2.128 ± 0.745	2.456 ± 0.877
	No	2.028 ± 1.640	43.503 ± 8.561	9.497 ± 2.582	2.101 ± 0.715	2.436 ± 0.935	2.244 ± 0.75	2.717 ± 0.968
Household registration	Urban	2.294 ± 2.308	43.676 ± 8.871	9.606 ± 2.442	2.118 ± 0.694	2.556 ± 0.899	2.235 ± 0.749	2.697 ± 0.920
	Rural	2.423 ± 1.998	43.466 ± 8.835	9.074 ± 2.559	2.041 ± 0.707	2.417 ± 0.871	2.138 ± 0.748	2.478 ± 0.944
Type of school	Undergraduate	2.311 ± 2.112	43.404 ± 8.637	9.604 ± 2.483	2.124 ± 0.687	2.554 ± 0.868	2.221 ± 0.761	2.704 ± 0.965
	Specialized	2.407 ± 2.266	43.836 ± 9.148	9.048 ± 2.507	2.028 ± 0.716	2.411 ± 0.913	2.152 ± 0.733	2.457 ± 0.877
Grade	Freshman	2.114 ± 2.182	41.955 ± 9.242	9.997 ± 2.545	2.217 ± 0.731	2.680 ± 0.939	2.246 ± 0.741	2.854 ± 0.944
	Sophomore	2.325 ± 2.314	44.215 ± 8.881	9.375 ± 2.409	2.055 ± 0.664	2.511 ± 0.901	2.169 ± 0.753	2.640 ± 0.954
	Junior	2.282 ± 1.928	44.074 ± 8.402	8.991 ± 2.537	2.012 ± 0.728	2.350 ± 0.832	2.208 ± 0.770	2.421 ± 0.863
	Senior	3.116 ± 2.266	44.554 ± 8.852	8.891 ± 2.297	2.029 ± 0.606	2.394 ± 0.808	2.088 ± 0.702	2.380 ± 0.917

### 3.2 Analysis of the relationship between extracurricular physical exercise time, core self-evaluation, and academic stress among college students

In order to further clarify the interrelationships among the variables, bivariate correlation analyses were conducted with college students' extracurricular physical exercise time, core self-evaluation scores, and academic stress scores. The results showed that there was a significant correlation between college students' extracurricular physical exercise time and core self-evaluation scores (*r* = 0.225, *p* < 0.01), academic stress scores (*r* = −0.317, *p* < 0.01), and between core self-evaluation scores and academic stress scores (*r* = −0.371, *p* < 0.01), which indicated that there was a definite correlation between the variables. The detailed results are shown in Table 2.

**Table 2:** Relationship among extracurricular physical exercise, academic stress, core self-evaluation of college students.

**Independent variable**	**Extracurricular physical exercise time**	**Core self-evaluation**	**Academic stress**
Extracurricular physical exercise time	1	0.225 (0.000)	−0.317 (0.000)
Core self-evaluation	0.225 (0.000)	1	−0.371 (0.000)
Academic stress	−0.317 (0.000)	−0.371 (0.000)	1

### 3.3 The mediating role of core self-evaluation in the relationship between extracurricular physical exercise and academic stress

In order to further clarify the specific mechanism of the relationship between extracurricular physical exercise and academic stress, this study added the core self-evaluation indicators and referred to the testing process of Wen Zhonglin and Ye Baojuan for the analysis of mediating roles. On the basis of controlling other variables, firstly, an unmediated model-equation 1 (*R*^2^= 0.155, *F* = 33.648) was established with academic stress as the dependent variable and extracurricular physical exercise as the independent variable, and the results showed a significant negative predictive effect (β = −0.306, *t* = −10.853, *P* = 0.000). Secondly, model-equation 2 (*R*^2^= 0.059, *F* = 11.432) was developed with core self-evaluation as the dependent variable and extracurricular physical exercise as the independent variable, and the results showed a positive predictive effect (β = 0.219, *t* = 7.365, *P* = 0.000). Finally, model-equation 3 (*R*^2^= 0.242, *F* = 50.055) was built with academic stress as dependent variable and core self-evaluation and extracurricular physical exercise as independent variables, and the results showed a negative predictive effect for both (β = −0.303, *t* = −11.209, *P* = 0.000), (β = 0.239, *t* = −8.750, *P* = 0.000). The detailed results are shown in Table 3.

**Table 3:** Mediation model test of academic stress among college students.

**Variant**	**Equation 1**			**Equation 2**			**Equation 3**		
	**(Academic stress)**			**(Core self-evaluation)**			**(Academic stress)**		
	β	***t***	***P***	β	***t***	***P***	β	***t***	***P***
Gender	0.212	3.457	0.001	−0.009	−0.148	0.882	0.199	3.598	0.000
Only child	−0.187	−3.322	0.001	0.039	0.656	0.512	−0.175	−3.283	0.001
Household registration	−0.078	−1.332	0.183	−0.075	−1.222	0.222	−0.101	−1.817	0.069
Type of school	−0.229	−3.968	0.000	0.073	1.202	0.229	−0.207	−3.778	0.000
Grade	−0.122	−4.168	0.000	0.083	2.703	0.007	−0.097	−3.474	0.000
Extracurricular physical exercise time	−0.306	−10.853	0.000	0.219	7.365	0.000	−0.239	−8.750	0.000
Core self-evaluation							−0.303	−11.209	0.000
*R^2^*	0.155			0.059			0.242		
*F*	33.648			11.432			50.055		

In the relationship between extracurricular physical exercise and academic stress, core self-evaluation played a partial mediating role (BootLLCI = −0.091, BootULCI = −0.046), and the mediating effect accounted for 21.9%. Meanwhile, the direct effect of extracurricular physical exercise accounted for 78.1%, which still had a high degree of direct predictive effect, indicating that extracurricular physical exercise not only acts directly on academic stress, but also indirectly through core self-evaluation. The specific effect values of the model are shown in Table 4.

**Table 4:** Specific values of each effect.

	**Efficiency value**	**Boot standard error**	**Boot CI standard lower limit**	**Boot CI standard ceiling**	**Relative effect value**
Aggregate effect	−0.306	0.028	−0.361	−0.251	
Direct effect	−0.239	0.027	−0.293	−0.186	78.1%
Intermediary effect	−0.067	0.011	−0.091	−0.046	21.9%

### 3.4 The mediating role of core self-evaluation in the relationship between extracurricular physical exercise and dimensions of academic stress

In order to further clarify which aspects of college students' core self-evaluation mediate academic stress, this study examined the mediating effects of “feeling difficult in learning”, “worrying about lagging behind other students in studies”, “not being able to keep up with the pace of study despite great effort”, and “worrying about exams”, etc. The results showed that the 95% confidence intervals do not include zero, and the mediating effect of core self-evaluation is highest in “worrying about lagging behind other students in studies” and “worrying about exams”, which account for 24.4% and 24.3% respectively. The detailed results are shown in Table 5.

**Table 5:** Test of mediating effects of various dimensions of academic stress.

	**Efficiency value**	**Boot standard error**	**Boot CI standard lower limit**	**Boot CI standard ceiling**	**Relative effect value**
Feeling difficult in learning	−0.047	0.001	−0.066	−0.030	19.9%
Worrying about lagging behind other students in studies	−0.052	0.010	−0.074	−0.033	24.4%
Not being able to keep up with the pace of study despite great effort	−0.054	0.010	−0.076	−0.036	20.3%
Worrying about exams	−0.052	0.011	−0.073	−0.032	24.3%

## 4 Discussion

Adolescents in a long-term stressful environment are prone to anxiety and depression and other psychological problems, and in serious cases, even self-inflicted suicidal tendencies (Blöte et al., 2015). Academic stress, as a diffuse source of pressure in college students, is a subjective feeling that exceeds their ability to cope with their studies, and permeates all aspects of their academic life. In daily life, if the academic stress is too big, it will affect physical and mental health, and will also cause a lack of concentration in learning, easy to produce persistent learning fatigue and anorexia, leading to a decline in academic performance (Nilani and Crystal, 2016). This study found that the overall academic stress of college students is already high, with about half of the students feeling pressure in the aspects of “worrying about lagging behind other students in studies” and “worrying about exams”, which indicates that there are more horizontal comparisons among students, and more students are worried about exams.

Among the many ways to relieve stress, physical exercise is a good way, especially compared with the fixed-time exercise in physical education class, the active form of extracurricular physical exercise can better reflect the subjective exercise willingness, and it is more likely to produce a good relaxation effect, but at present the research on the direct (Wilson-Salandy and Nies, 2012; Fares et al., 2015) relationship between extracurricular physical exercise and academic stress is still relatively rare. In the newly amended Sports Law of the People's Republic of China, it is clearly stated that students should exercise at school for not <1 h a day. However, the current situation of college students' participation in physical exercise is worrying (Wu, 2017). In this study, it is also found that the weekly extracurricular physical exercise time of college students is obviously less, more than 80% of the students have less than half an hour of extracurricular exercise per day on average, and a certain proportion of them hardly exercise, which indicates that the independent exercise of the students is generally less, and their awareness of exercise is weak, and the phenomenon of sedentary lifestyle has become a common phenomenon (Hoare et al., 2016). This will have a negative impact on their physical health and mental health (Lurati, 2018). After controlling for the above categorical variables, this study found that there is a significant negative relationship between extracurricular physical exercise time and academic stress, and that increasing extracurricular physical exercise time can help to reduce college students' academic stress. In order to further clarify the mechanism of this relationship, we conducted an in-depth enquiry on the basis of the previous study.

Extracurricular physical exercise has an obvious direct effect on academic stress. Since the source of students' academic stress mainly comes from the pressure of the individual's internal cognition of learning and the pressure of external stimuli felt in the process of learning, it can be seen through the analysis of the stress interaction model that learning itself does not produce stress, and the cognitive interpretation of learning and the situation is the root cause. Therefore, peer relationships and a good exercise environment in extracurricular physical exercise can help to resolve negative emotions (Fraser-Thomas and Côté, 2009), especially after an event or a negative state of mind, it helps to mobilize ruminative thinking and to get out of a bad situation as quickly as possible, while the positive experience of exercising contributes to the development of the psychosocial aspect, and the enjoyment generated by these exercises helps to reduce the pressure of external stimuli in the learning process. In addition, there is a strong relationship between physical exercise and cognitive ability (Gomez-Pinilla and Hillman, 2013). Active extracurricular exercise can effectively relieve the fatigue caused by overuse of the brain, promote beneficial changes in the brain. It also improves students' memory capacity, enhances self-efficacy in learning, stimulates positive feelings of focusing on thinking in learning, changes the understanding of learning in negative situations, and helps to reduce the internal pressure generated in the process of learning (Tomporowski and Ellis, 1986). Students' extracurricular physical exercise mostly belongs to autonomous sports, and there is a higher correlation between it and the alleviation of academic stress, so extracurricular physical exercise plays a more important role in alleviating the academic stress of college students nowadays. These may be important reasons for the direct effect of extracurricular physical exercise (Cavicchiolo et al., 2022).

The core self-evaluation of college students plays an obvious indirect role. Core self-evaluation is an important result of personality research in recent years, which mainly involves self-esteem, self-efficacy, emotional stability, and sources of psychological control. According to the frame-of-reference theory, students' core self-evaluation in the process of growing up has an important relationship with the overall school environment and academic performance (Diotaiuti et al., 2021). The school's exercise environment is also closely related to students' core self-evaluation, and sports can significantly increase students' self-esteem and self-confidence (Graydon, 1997), and positively predicting self-efficacy, and increased recognition of one's own abilities, which to a certain extent helps to reduce excessive anxiety and worry about academics, and is conducive to reducing academic stress (Pujol-Cols and Lazzaro-Salazar, 2020). The explanatory role of cognition is more important in studies on sources of control. Physical exercise has a significant positive correlation with the dimensions of internal control type (Lynn et al., 1969), and regular participation in extracurricular physical exercise helps to increase the level of core self-evaluation, while students with high core self-evaluation may be more inclined to regard academic stress as a challenge rather than a threat, and when experiencing stressful setbacks can produce a more positive state of mind to face the stress, and the corresponding depression or somatic symptoms will be lower, and they are able to maintain a higher level of psychological resilience to stress that alleviated their negative feelings about academic stress, which in turn helped to reduce academic stress (Sun, 2015). The role of regulation is more important in studies of emotional stabilization. It has been pointed out that different sports programs can make obvious differences in emotional stability, and sports can play an obvious role in the regulation of negative emotions and keep themselves in a positive and optimistic state of mind (Vorontsova-Wenger et al., 2022); at the same time, students with a high core self-evaluation tend to have more positive emotion regulation strategies, such as emotional expression, emotional confession, etc., which can help to alleviate negative emotions, and this positive mechanism of emotion regulation helps individuals to better cope with academic stress, reduce anxiety and worry about academics, and show lower levels of academic stress and academic burnout. In addition, students' core self-evaluation belongs to the individual's high-level personality factors, the level of self-evaluation will be affected by a variety of environmental factors in the school, and the influence of the formation of the physical exercise environment is not limited to the level of self-esteem, self-efficacy, emotional stability, and sources of psychological control, but also need to be combined with other factors to explore the deeper interrelationships. Taken together, these aspects suggest that active extracurricular physical exercise can indirectly reduce academic stress by increasing core self-evaluation levels. This study also found that more students felt academic stress in the areas of “worrying about lagging behind other students” and “worrying about exams”, and the indirect effect of core self-evaluation also played an important role in this area, accounting for 24.4% and 24.3% of the total effect of extracurricular physical exercise, which also suggests that extracurricular physical exercise is more effective in reducing the source of stress for more students and more related to the mediating effect of core self-evaluation.

In addition to improving cardiorespiratory fitness and reducing the incidence of obesity among college students, regular extracurricular physical exercise positively contributes to students' core self-evaluation by boosting self-efficacy, increasing positive affective experiences, building a sense of accomplishment, and fostering resilience, as well as enhancing social support and identity (Yao et al., 2023). This impact contributes to an individual's psychological wellbeing, enhances quality of life, and also enables individuals to cope more positively with various life challenges. These good benefits of exercise have long been confirmed in a large number of studies, however, in this study, it was found that the amount of physical exercise of college students is obviously insufficient, and the academic stress of the students is significantly higher, whether for physical health or psychological health, college students should be encouraged to go into the sports field, and active extracurricular physical exercise should be given enough attention by college students.

Conclusion of this study: Currently, college students have different degrees of academic stress; extracurricular physical exercise can reduce academic stress through direct effects, and also through the indirect effect of improving the level of core self-evaluation, and active extracurricular physical exercise has become an important way to promote students' physical and mental health.

Limitations of this study: the study mainly points out the significant relationship between college students' academic stress and extracurricular physical exercise, as well as the mediating effect of the core self-evaluation, and fails to carry out a lag analysis from the perspective of time, but it can provide a theoretical basis for subsequent in-depth investigation. The sample selected in the study still has some limitations, and it was not clear whether there was a different relationship between factors such as sport, intensity and amount of exercise and academic stress, and these could be considered in the future using a more integrated approach to research or by adding some assessment questions.

## Data availability statement

The original contributions presented in the study are included in the article/supplementary material, further inquiries can be directed to the corresponding author.

## Ethics statement

The questionnaire of this study was approved by the Ethical Approval of the Academic Committee of the Department of Physical Education of Nanjing University of Science and Technology (TYB-2023001) and informed consent was obtained from all the investigated students.

## Author contributions

GZ: Data curation, Formal analysis, Methodology, Project administration, Supervision, Validation, Writing—original draft. WC: Conceptualization, Data curation, Investigation, Software, Supervision, Writing—original draft. YY: Formal analysis, Project administration, Validation, Writing—original draft. SB: Conceptualization, Data curation, Funding acquisition, Investigation, Methodology, Resources, Supervision, Visualization, Writing—review & editing.
